# Prevalence of neutralizing autoantibodies against type I interferon in dengue patients

**DOI:** 10.70962/jhi.20260072

**Published:** 2026-09-01

**Authors:** Mengtian Yang, Yixin Liao, Xiaorong Yang, Jiaqi Fan, Min Liao, Yongchang Wu, Huiqin Yang, Jian Wang, Wei Zhang, Feng Li, Haisheng Yu, Yun Ling

**Affiliations:** 1 Institute of Infectious Diseases, Guangzhou Eighth People’s Hospital, Guangzhou Medical University, Guangzhou, China; 2 Guangzhou Key Laboratory of Clinical Pathogen Research for Infectious Diseases, Guangzhou, China; 3 Department of Infectious Disease, Shanghai Public Health Clinical Center, Fudan University, Shanghai, China

## Abstract

The presence of neutralizing autoantibodies (NAbs) against type I IFN (IFN-I) has been associated with severe complications arising from various viral infections. However, the prevalence of IFN-I NAbs in dengue virus (DENV) infection remains unclear. Here, we enrolled 216 hospitalized dengue patients in 2024, including 7 individuals with dengue shock syndrome (DSS) and 42 individuals with dengue hemorrhagic fever (DHF). Utilizing a sensitive luciferase reporter assay, IFN-I NAbs were detected in 7 patients (14.3% of DSS/DHF cases), and their neutralizing ability was further validated in DENV-infected cells. Notably, all NAb-positive patients exhibited plasma leakage tendency and cardiac injury and were significantly older than the overall cohort (75.34 vs. 50.75, P *=* 0.010). IFN-I NAbs were correlated with clinical parameters of myocardial injury and plasma leakage, with increased odds ratio (OR) for cardiac injury (OR = 22.8, P = 0.003) and hypoproteinemia (OR = 53.5, P = 0.001). These findings suggest that IFN-I NAbs may contribute to severe clinical manifestations of dengue, warranting validation in larger cohorts.

## Teaser

The presence of type I IFN-neutralizing antibodies (NAbs) is a life-threatening risk factor for multiple infectious diseases. This study reports the prevalence of NAbs in dengue patients and indicates that patients with NAbs may have a higher risk of cardiac injury.

## Introduction

Dengue virus (DENV), primarily transmitted by *Aedes aegypti* and *Aedes albopictus* mosquitoes, causes acute dengue fever (DF), characterized by a broad clinical spectrum from mild, self-limiting illness to life-threatening complications. Historically confined to tropical and subtropical regions, DENV transmission has expanded globally over the past two decades. It is estimated that ∼390 million DENV infections occur annually worldwide, with about 96 million individuals manifesting clinical symptoms and 22,000 fatalities (1, 2). Although most infected patients experience merely mild symptoms, ∼5% develop severe life-threatening complications, such as severe plasma leakage, shock, and severe organ injury (3). Mortality in dengue patients is primarily attributed to shock, encephalitis, and myocarditis. Established risk factors for severe disease include young age, pregnancy, advanced age, diabetes, hypertension, and pre-existing cardiac or renal conditions (4). Nevertheless, the roles of immune and genetic factors in modulating disease progression remain inadequately understood, warranting further investigation into their contributions to dengue pathogenesis.

Type I IFNs (IFNs-I) are integral to antiviral immunity, primarily through the induction of IFN-stimulated genes. Firstly, multiple inherited genetic mutations that could cause a compromised or deficient IFN pathway have been identified. Key mutations in the IFN receptor subunits, *IFNAR1* and *IFNR2*, reduce their binding affinity to IFN-I, resulting in the host being unresponsive to IFN. Children with *IFNAR1/IFNR2* deficiency developed encephalitis, as well as liver and kidney dysfunction, after receiving either the live-attenuated measles, mumps, and rubella vaccine or the live-attenuated yellow fever vaccine, with some cases resulting in death (5, 6). Similarly, individuals with mutated *STAT1/2*, the key regulator downstream of the IFN pathway, are prone to develop fatal encephalitis, pneumonia, hepatitis, and severe varicella after being infected with various viruses such as HSV, enterovirus (EV), EBV, CMV, adenovirus (AdV), and Influenza A virus (IAV) (7, 8, 9, 10, 11). *TYK2*-deficient patients also suffered from severe pneumonia, encephalitis, lymphadenitis, and recurrent oral herpes after infection (12, 13, 14). Secondly, hosts could develop IFN-I–neutralizing autoantibodies (NAbs) for some unknown reason, which compromises the binding of IFN to their receptors, mimicking a non-inherited but acquired IFN-I pathway deficiency. IFN-I NAbs are detected in <0.2% of the general population but increase to 4–7% among individuals aged 70 years or older (15, 16, 17, 18). The NAbs have been more frequently identified in viral infected patients, for example, West Nile virus (WNV) encephalitis (40%) (19, 20), HSV-triggered fulminant viral hepatitis (37.5%) (21), severe COVID-19 pneumonia (11.8–25%) (22, 23, 24, 25, 26), critical Middle East respiratory syndrome pneumonia (25%) (27), severe tick-borne encephalitis (10%) (28), severe influenza pneumonia (5%) (29, 30), and most cases of the rarer severe Usutu virus (USUV) (2 of 3, 66.6%), Powassan virus (POWV) (a single severe case), and Ross River virus (the sickest patients) diseases (31). Moreover, NAbs predispose individuals to severe adverse reactions following administration of the yellow fever live-attenuated vaccine (30%) (32). In severe cases of multiple flavivirus (WNV, tick-borne encephalitis virus, POWV, and USUV) infections, NAb positivity shows a strong correlation with clinical disease severity.

However, the role of IFN-I NAb in dengue infection remains uninvestigated. We retrospectively enrolled 216 dengue patients of varying severity in 2024, including 7 cases of dengue shock syndrome (DSS), 42 cases of dengue hemorrhagic fever (DHF), and 167 cases of non-severe DF, and identified 7 NAb-positive individuals—accounting for 14.3% of the combined DSS and DHF groups. The NAbs against IFN-α2 and/or IFN-ω are mainly detected in the older patients and are associated with increased disease severity in dengue, particularly a higher risk of plasma leakage and cardiac injury during acute DENV infection.

## Results

### Demographic and clinical characteristics of dengue patients

**Figure 1.**
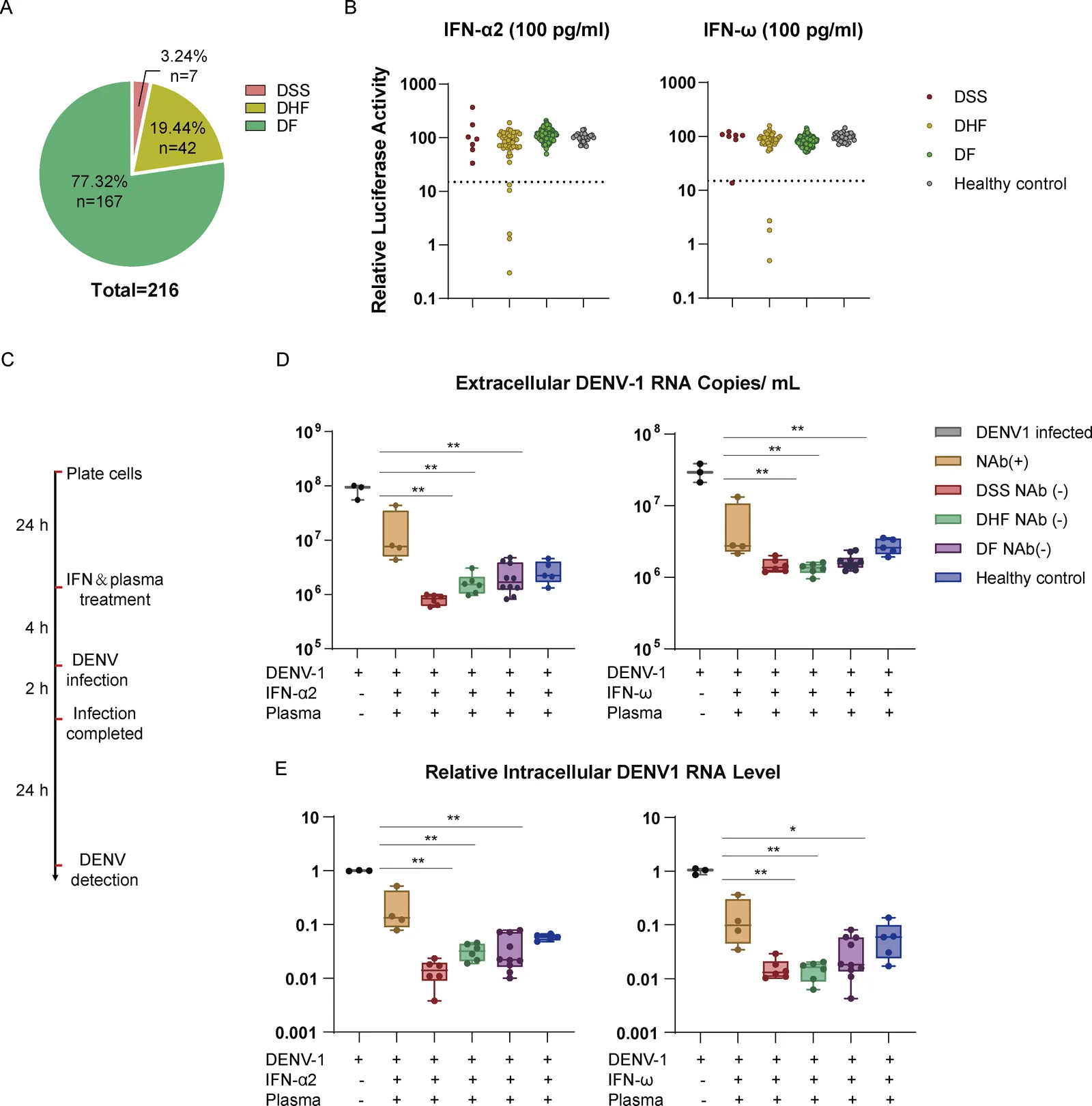
**Prevalence of IFN-α2 and/or IFN-ω NAbs in dengue patients. (A)** The proportion of patients in the DSS, DHF, and DF groups. **(B)** The neutralization of IFN-α2 (left) and IFN-ω (right) in the presence of plasma from dengue patients (DSS, DHF, and DF groups) or healthy individuals (healthy control group, *N* = 29) by detecting IRSE-promoter luciferase activity. The relative luciferase activity was normalized by *Renilla*. The dotted line in panel B indicates the 15% cutoff, with values below 15% considered positive for neutralizing activity. NAb-negative samples were tested once or twice, whereas NAb-positive samples were tested three times to confirm the results. **(C)** Experimental scheme of D and E. **(D and E)** The inhibitory effect of dengue patients’ plasma on IFN-regulated antiviral activity in Huh7 cells. The DENV-1 RNA copies in extracellular (D) and intracellular (E). The DENV-1 RNA levels in intracellular were normalized by GAPDH. Statistical analysis was performed using the Mann–Whitney U test. The experiment was independently repeated three times. The asterisks indicate statistical significance: *P < 0.05 and **P < 0.01.

This study included 216 dengue patients admitted to the hospital from September to December 2024 in Guangzhou, China. To mitigate potential confounding attributable to viral serotype, patients with diagnosed infections other than the DENV-1 serotype were excluded from the cohort. Among our cohort, 192 (88.9%) were tested DENV-1 RNA positive; for the remaining 24 patients who tested positive for NS1 antigen or dengue-specific IgM antibodies, the quantitative RT-PCR (qRT-PCR) result for DENV RNA was undetermined. The patients were categorized into three groups based on the clinical manifestation severity: DSS (*N* = 7), DHF (*N* = 42), and DF (*N* = 167) (Fig. 1 A and Table 1). Notably, there were three patients experienced secondary DENV infection, one of whom progressed to DSS, while the other two developed DHF (Table 1). Significant differences were identified in the complications across the three groups, including hypoproteinemia, shock, kidney injury, cardiac injury, and cerebral injury, as well as in the use of therapeutic interventions: glucocorticoids, platelet transfusion, and albumin (ALB) infusion (Table 1). Consistent with expectations, patients in the DSS group had the longest median hospitalization duration compared with the other two groups (8.00/6.89/5.78, P < 0.001).

**Table 1.** Demographics and clinical characteristics of patients on admission

​	Overall	DSS	DHF	DF	P
**Demographic**					
*N*	216	7	42	167	​
Age (median [IQR])	50.75 [32.97–61.61]	67.00 [52.64–78.35]	61.72 [52.02–70.88]	46.97 [29.58–57.26]	<0.001
0–49 (%)	103 (47.7)	2 (28.6)	7 (16.7)	94 (56.3)	​
50–59 (%)	49 (22.7)	1 (14.3)	10 (23.8)	38 (22.8)	​
60–69 (%)	36 (16.7)	1 (14.3)	12 (28.6)	23 (13.8)	​
70–79 (%)	21 (9.7)	1 (14.3)	10 (23.8)	10 (6.0)	​
80+ (%)	7 (3.2)	2 (28.6)	3 (7.1)	2 (1.2)	​
Sex (male [%])	123 (56.9)	4 (57.1)	17 (40.5)	102 (61.1)	0.058
Secondary dengue infection (%)	3 (1.4)	1 (14.3)	2 (4.8)	0	0.006
**Comorbidities**					
Hypertension (%)	34 (15.7)	1 (14.3)	10 (23.8)	23 (13.8)	0.241
Diabetes (%)	20 (9.3)	0	9 (21.4)	11 (6.6)	0.016
Heart disease (%)	12 (5.6)	0	5 (11.9)	7 (4.2)	0.163
Renal disease (%)	12 (5.6)	1 (14.3)	6 (14.3)	5 (3.0)	0.005
Malignant tumor (%)	5 (2.3)	0	4 (9.5)	1 (0.6)	0.018
Neuropsychopathy (%)	4 (1.9)	0	3 (7.1)	1 (0.6)	0.041
**Complication**					
Hypoproteinemia (%)	67 (31.0)	7 (100)	39 (92.9)	21 (12.6)	<0.001
Shock (%)	5 (2.3)	5 (71.4)	0	0	<0.001
Hepatic injury (%)	126 (58.3)	6 (85.7)	28 (66.7)	92 (55.1)	0.134
Kidney injury (%)	21 (9.7)	6 (85.7)	8 (19.0)	7 (4.2)	<0.001
Cardiac injury (%)	53 (24.5)	5 (71.4)	15 (35.7)	33 (19.8)	0.002
Cerebral injury (%)	17 (7.9)	3 (42.9)	6 (14.3)	8 (4.8)	0.002
**Treatment**					
Rehydration (%)	203 (94.0)	7 (100)	40 (95.2)	156 (93.4)	1.000
Glucocorticoids (%)	13 (6.0)	3 (42.9)	5 (11.9)	5 (3.0)	<0.001
Platelet transfusion (%)	32 (14.8)	4 (57.1)	14 (33.3)	14 (8.4)	<0.001
ALB infusion (%)	18 (8.3)	7 (100)	9 (21.4)	2 (1.2)	<0.001
Days of hospitalization (median [IQR])	5.89 [4.66–7.78]	8.00 [6.95–22.00]	6.89 [5.05–9.91]	5.78 [4.54–7.37]	<0.001

Data are reported as *N*, *N* (%), or median [IQR]. Chi-square test or Fisher’s exact test was used to analyze the effect of dichotomous variables, and a Kruskal–Wallis test was used for continuous variables. A P value <0.05 was considered to indicate statistical significance. IQR, interquartile range.

### NAbs against IFN-α2 and/or IFN-ω in dengue patients

**Figure S1.**
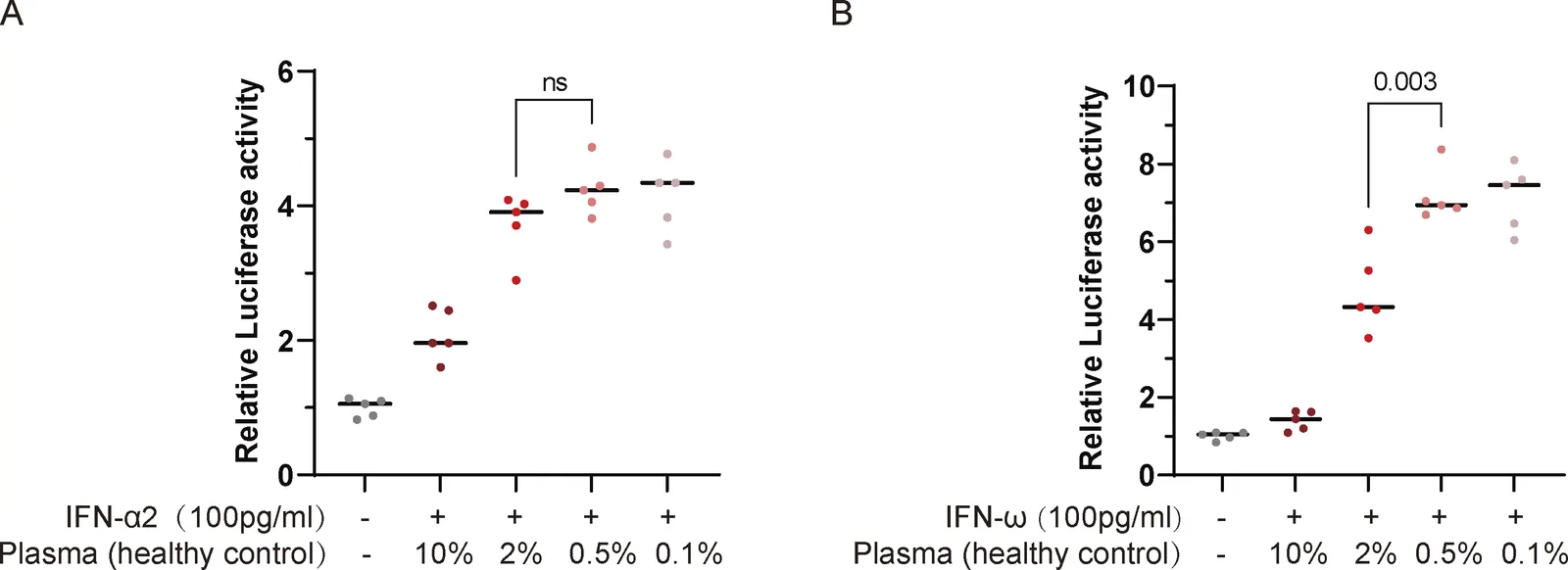
**Effects of different proportions of plasma (collected by EDTA-anticoagulated tubes) on IFN-I activation. (A and B)** Effects of different proportions of plasma (collected by EDTA-anticoagulated tubes from healthy adults without NAbs) on IFN-α2 (A) and IFN-ω (B) activation.

The presence of NAbs against IFN-α2 and IFN-ω was evaluated in plasma samples derived from our cohort, using a previously described sensitive luciferase reporter assay (19, 26). Since we collected whole blood samples using EDTA-anticoagulated tubes, we carefully considered the potential influence of residual EDTA on IFN activation in plasma. To address this concern, we conducted a dedicated experiment using plasma from healthy human donors (who were confirmed to be negative for IFN NAbs). This plasma was tested across a range of dilution factors, and based on our results (Fig. S1), the optimal dilutions were determined to be 2:100 (2%) for IFN-α2 and 1:100 (1%) for IFN-ω. Given that most previous studies assess neutralization using either 10% plasma with 100 pg/ml (low concentration) or 10% plasma with 10 ng/ml (high concentration) of IFN-I, we selected a more intermediate ratio: 1–2% plasma with 100 pg/ml IFN-I. Among the 216 dengue patients enrolled, IFN-I NAbs were detected in the plasma of seven individuals (3.2%, 95% confidence interval [CI] [1.6–6.5]). Specifically, three patients had NAbs against IFN-α2 only, two against IFN-ω only, and two were positive for both. Importantly, one of NAb against IFN-ω–positive cases belonged to the DSS group (1 of 7, 14.3%, 95% CI [2.6–51.3]), and the other NAb-positive cases belonged to the DHF group (6 of 42, 14.3%, 95% CI [6.7–27.9]) (Fig. 1 B and Table 3).

IFN-I has been documented to possess strong antiviral properties against DENV. To assess the impact of IFN-I NAbs on antiviral efficacy, Huh7 cells were infected with a clinical DENV-1 strain (detailed in Fig. 1 C). Notably, a patient positive for anti-IFN-α NAbs (a 78-year-old female in the DHF group) experienced a secondary DENV infection and was found to have dengue-specific IgG during the early stage of infection. Her plasma exhibited robust antiviral activity against DENV in vitro. Consequently, this patient was excluded from subsequent analyses. As anticipated, the NAb(+) group exhibited the highest levels of viral replication among the pretreatment groups, with the exception of the aforementioned secondary-infection patient. Viral replication was measured by both extracellular and intracellular DENV RNA levels (Fig. 1, D and E). Interestingly, DENV replication was slightly reduced in patients with NAb(−) compared with healthy controls, suggesting the presence of additional antiviral factors in the plasma of dengue patients. Collectively, these findings indicate that IFN-I NAbs are restricted to the DSS and DHF group and can impair IFN-1–mediated antiviral responses in dengue patients.

### Association of age with the prevalence of NAbs against IFN-I in dengue patients

Following the identification of patients with IFN-I NAbs, we conducted a comprehensive comparison of clinical characteristics between NAb(+) and NAb(−) patients (Tables 2 and 3).

**Table 2.** Comparing demographic and clinical characteristics of dengue patients with and without IFN-I NAbs

​	Overall	NAb(+)	DSS NAb(−)	DHF NAb(−)	DF NAb(−)	P (NAb(+) group compared with the other groups)
**Demographic**						
*N*	216	7	6	36	167	​
Age (median [IQR])	50.75 [32.97–61.61]	75.34 [54.16–81.90]	70.50 [59.71–80.53]	61.36 [51.04–69.39]	46.97 [29.58–57.26]	1.000/1.000/0.007
0–49 (%)	103 (47.7)	1 (14.3)	1 (16.7)	7 (19.4)	94 (56.3)	​
50–59 (%)	49 (22.7)	2 (28.6)	1 (16.7)	8 (22.2)	38 (22.8)	​
60–69 (%)	36 (16.7)	0	1 (16.7)	12 (33.3)	23 (13.8)	​
70–79 (%)	21 (9.7)	2 (28.6)	1 (16.7)	8 (22.2)	10 (6.0)	​
80+ (%)	7 (3.2)	2 (28.6)	2 (33.3)	1 (2.8)	2 (1.2)	​
Sex (male [%])	123 (56.9)	3 (42.9)	4 (66.7)	14 (38.9)	102 (61.1)	0.592/1.000/0.437
Secondary dengue infection (%)	3 (1.4)	1 (14.3)	1 (16.7)	1 (2.8)	0	1.000/0.302/0.040
**Comorbidities**						
Hypertension (%)	34 (15.7)	3 (42.9)	1 (16.7)	7 (19.4)	23 (13.8)	0.559/0.325/0.069
Diabetes (%)	20 (9.3)	2 (28.6)	0	17 (47.2)	11 (6.6)	0.462/0.437/0.088
Heart disease (%)	12 (5.6)	2 (28.6)	0	3 (8.3)	7 (4.2)	0.462/0.180/0.044
Renal disease (%)	12 (5.6)	0	1 (16.7)	6 (16.7)	5 (3.0)	1.000/0.567/1.000
Malignant tumor (%)	5 (2.3)	0	0	4 (11.1)	1 (0.6)	1.000/1.000/1.000
Neuropsychopathy (%)	4 (1.9)	0	0	3 (8.3)	1 (0.6)	1.000/1.000/1.000
**Complication**						
Hypoproteinemia (%)	67 (31.0)	7 (100)	6 (100)	33 (91.7)	21 (12.6)	1.000/1.000/<0.001
Shock (%)	5 (2.3)	1 (14.3)	4 (66.7)	0	0	0.266/0.163/0.040
Hepatic injury (%)	126 (58.3)	5 (71.4)	5 (83.3)	24 (66.7)	92 (55.1)	1.000/1.000/0.466
Kidney injury (%)	21 (9.7)	3 (42.9)	5 (83.3)	6 (16.7)	7 (4.2)	0.592/0.147/0.004
Cardiac injury (%)	52 (24.1)	7 (100)	4 (66.7)	8 (22.2)	33 (19.8)	1.000/<0.001/<0.001
Cerebral injury (%)	17 (7.9)	0	3 (50.0)	6 (16.7)	8 (4.8)	0.192/0.567/1.000
**Treatment**						
Rehydration (%)	203 (94.0)	7 (100)	6 (100)	34 (94.4)	156 (93.4)	1.000/1.000/1.000
Glucocorticoids (%)	13 (6.0)	1 (14.3)	2 (33.3)	5 (13.9)	5 (3.0)	0.443/1.000/0.221
Platelet transfusion (%)	32 (14.8)	6 (85.7)	3 (50.0)	9 (25.0)	14 (8.4)	0.266/0.005/<0.001
ALB infusion (%)	18 (8.3)	3 (42.6)	6 (100)	7 (19.4)	2 (1.2)	0.266/0.325/<0.001
Days of hospitalization (median [IQR])	5.89 [4.66–7.78]	6.00 [5.41–6.93]	11.00 [7.17–26.00]	7.18 [5.30–9.99]	5.78 [4.54–7.37]	0.412/1.000/1.000

Data are reported as *N*, *N* (%), or median [IQR]. Fisher’s exact test was used to analyze the effect of dichotomous variables, and a Kruskal–Wallis test was used for continuous variables. A P value <0.05 was considered to indicate statistical significance. IQR, interquartile range.

**Table 3.** Characteristics of dengue patients with IFN-neutralizing effects

No. and group	Sex	Age (years)	DENV serotype	NAbs	Outcomes	Symptoms	Hemorrhage-related parameters			Organ injury or abnormality			
							PLT (×10^9^/L)	HB (g/L)	HCT (%)	Cardiac	Hepatic	Kidney	Lung
P1 (DHF)	M	53	DENV-1	IFN-α2	Survival	Thrombocytopenia, mucosal bleeding	57	145	44.2	++ Myocardial damage	++	–	–
P2 (DHF)	F	78	DENV-1	IFN-α2	Survival	Hypoalbuminemia, palpitation	93	79	24	++ Heart failure, myocarditis, and paroxysmal atrial fibrillation	–	+	–
P3 (DHF)	M	86	DENV-1	IFN-α2	Survival	Diarrhea, mucosal bleeding	77	115	34.5	+ Sinus bradycardia, aortic regurgitation, and left ventricular diastolic dysfunction	++	–	+
P4 (DHF)	M	55	Unknown	IFN-ω	Survival	Abdominal discomfort, epistaxis, and thrombocytopenia	17	128	38.5	+ Mitral regurgitation, and tricuspid regurgitation	++	–	–
P5 (DSS)	F	48	DENV-1	IFN-ω	Death	Thrombocytopenia, abdominal pain, diarrhea, and septic shock	13	40	<15	++ Myocarditis, paroxysmal atrial fibrillation	+	+	++
P6 (DHF)	F	75	DENV-1	IFN-α2/IFN-ω	Survival	Thrombocytopenia, abdominal discomfort	14	123	38.2	++ Heart failure, paroxysmal atrial fibrillation	+	+	–
P7 (DHF)	F	85	DENV-1	IFN-α2/IFN-ω	Survival	Abdominal pain, thrombocytopenia	59	91	29.8	++ Myocardial damage	–	–	–

P1–P3, the anti-IFN-α2 NAb group; P4 and P5, the anti-IFN-ω NAb group; and P6 and P7, the anti-IFN-α2 and anti-IFN-ω NAb group. F, female; M, male; PLT, platelet; HCT, hematocrit. +, organ dysfunction (hepatic/renal/pulmonary dysfunction, cardiac arrhythmia, etc.). ++, organ inflammation or injury (toxic hepatitis, myocarditis, myocardial injury, pneumonia, etc.).

**Figure 2.**
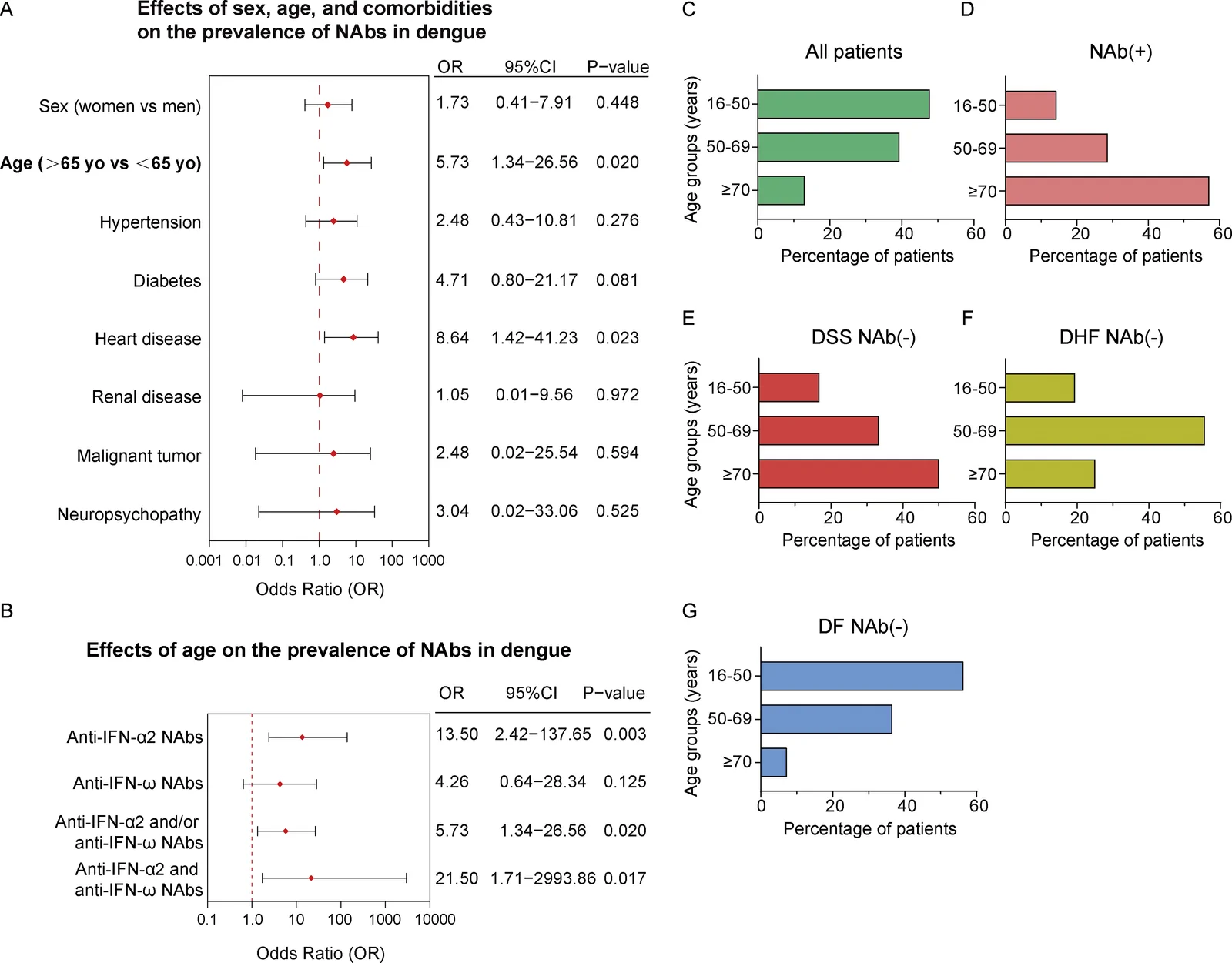
**Association of age with the prevalence of IFN-I NAbs in dengue patients. (A)** Effects of sex, age, and comorbidities on the prevalence of NAbs in dengue patients. Firth’s bias-corrected logistic regression was used to assess the association between clinical characteristics (sex, age, and comorbidities) and NAbs (defined as the combined anti–IFN-α2 NAbs and anti–IFN-ω NAbs group) with adjusted ORs and 95% CIs. A P value <0.05 was considered statistically significant. “yo” is an abbreviation for “years old.” **(B)** Effects of age on the prevalence of IFN-I NAbs in dengue patients. Firth’s bias-corrected logistic regression was used to assess the association between age and IFN-I NAbs (analysis of age with anti–IFN-α2 NAbs, anti–IFN-ω NAbs, anti–IFN-α2 and/or anti–IFN-ω NAbs, anti–IFN-α2, and anti–IFN-ω NAbs, respectively). Adjusted ORs with 95% CIs were calculated, and a P value <0.05 was considered statistically significant. **(C–G)** Age distribution of individuals in the all patients (C), NAb(+) (D), DSS NAb(−) (E), DHF NAb(−) (F), and DF NAb(−) (G)groups.

We investigated the effects of sex, age, and multiple comorbidities (pre-existing underlying disease history prior to DENV infection) on the prevalence of NAbs in dengue using a Firth’s bias-corrected logistic regression analysis. The prevalence of NAbs was significantly higher in patients older than 65 years (odds ratio [OR] = 5.73, 95% CI: 1.34–26.56, P = 0.020) (Fig. 2 A), while no significant association was observed among patients harboring NAbs against IFN-ω (Fig. 2 B), which might be attributable to the small sample size in this subgroup. 85.7% (6 of 7) of NAb(+) patients were older than 50 years, but the majority of individuals in the entire cohort were under 50 years of age (Fig. 2, C–G). Notably, our cohort showed that the incidence of NAbs in dengue patients was also related to comorbidities of heart disease and independent of sex (Fig. 2 A), which was different from the higher incidence of IFN-I NAbs in male patients reported previously (22, 29).

### IFN-I NAbs were associated with increased dengue severity

**Figure 3.**
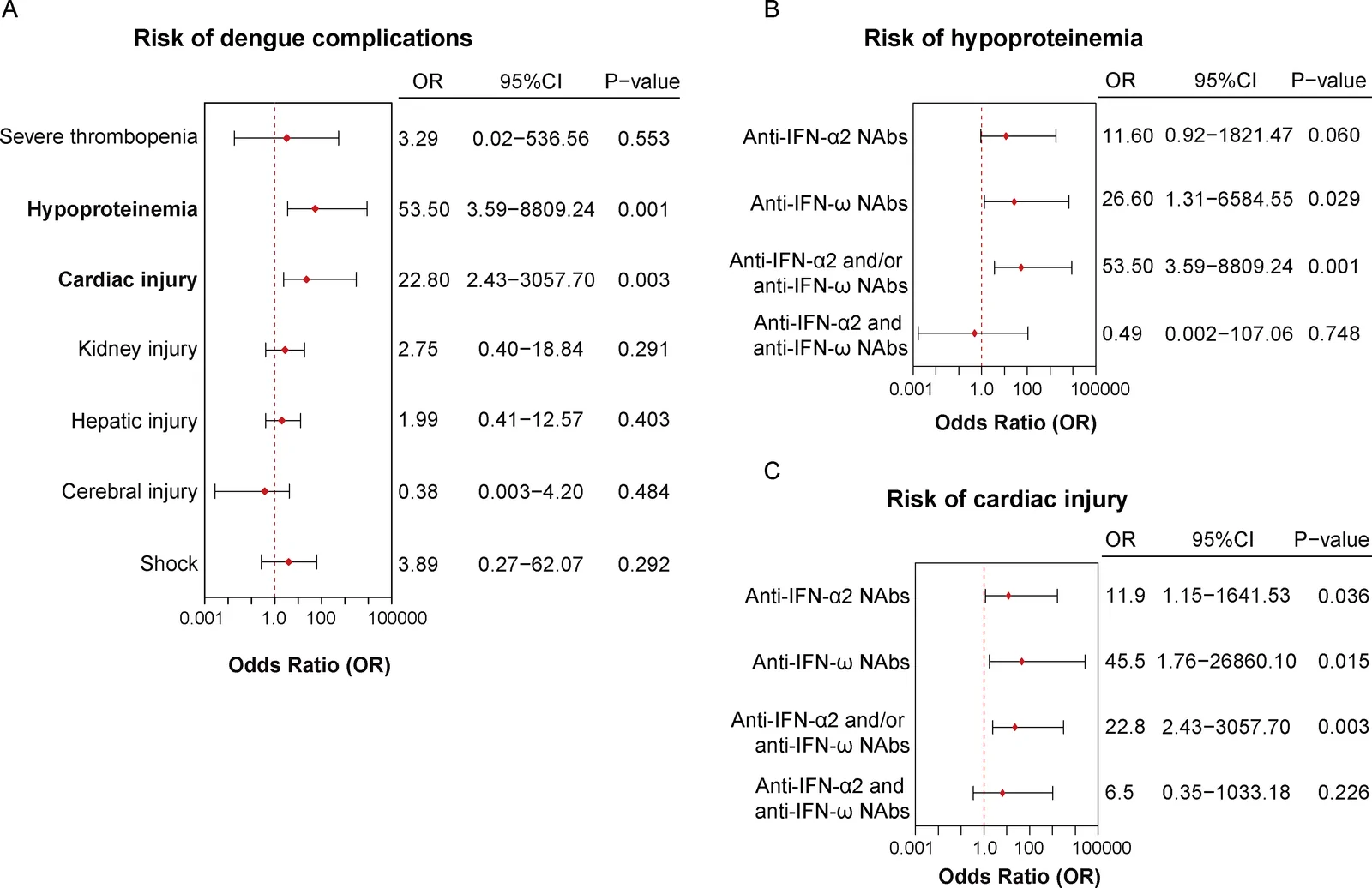
**Assessment of IFN-I autoantibodies as a potential risk factor for multiple dengue complications. (A)** The association between NAbs (defined as the combined anti–IFN-α2 NAbs and anti–IFN-ω NAbs group) and multiple complications. **(B)** The association between NAbs (anti–IFN-α2 NAbs, anti–IFN-ω NAbs, anti–IFN-α2 and/or anti–IFN-ω NAbs, anti–IFN-α2, and anti–IFN-ω NAbs, respectively) and hypoproteinemia. **(C)** The association between NAbs (anti–IFN-α2 NAbs, anti–IFN-ω NAbs, anti–IFN-α2 and/or anti–IFN-ω NAbs, anti–IFN-α2, and anti–IFN-ω NAbs, respectively) and cardiac injury. ORs and P values were estimated using Firth’s bias-corrected logistic regression, with adjustment for age and sex. Propensity score matching was also applied to adjust for age, sex, and comorbidity confounders.

The complication (acute disease onset following DENV infection) of dengue may progress gradually to hemorrhage, plasma leakage, multiple organ injuries, and shock. Notably, the incidence of most complications in NAb(+) patients was comparable with that in the DSS NAb(−) group, and higher than that in other NAb(−) counterparts, particularly for hypoproteinemia (which is a sign of plasma leakage) and cardiac injury, both of which were observed in all NAb(+) cases (Table 2). Similarly, the utilization of therapeutic interventions—especially platelet transfusions (85.7%)—was significantly greater compared with the other NAb(−) group (Table 2). These findings suggest that NAbs may be associated with more severe clinical manifestations during DENV infection. Using propensity score matching to mitigate confounding factors such as age, sex, and comorbidities (including heart related comorbidities), Firth’s bias-corrected logistic regression analyses conformed that the presence of NAbs exacerbate the risk of hypoproteinemia (OR = 53.50, 95% CI: 3.59–8809.24, P = 0.001) and cardiac injury (OR = 22.80, 95% CI: 2.43–3057.70, P = 0.003) (Fig. 3, A–C), and these complications were linked to disease progression toward more severe dengue diseases (DHF and DSS).

**Figure 4.**
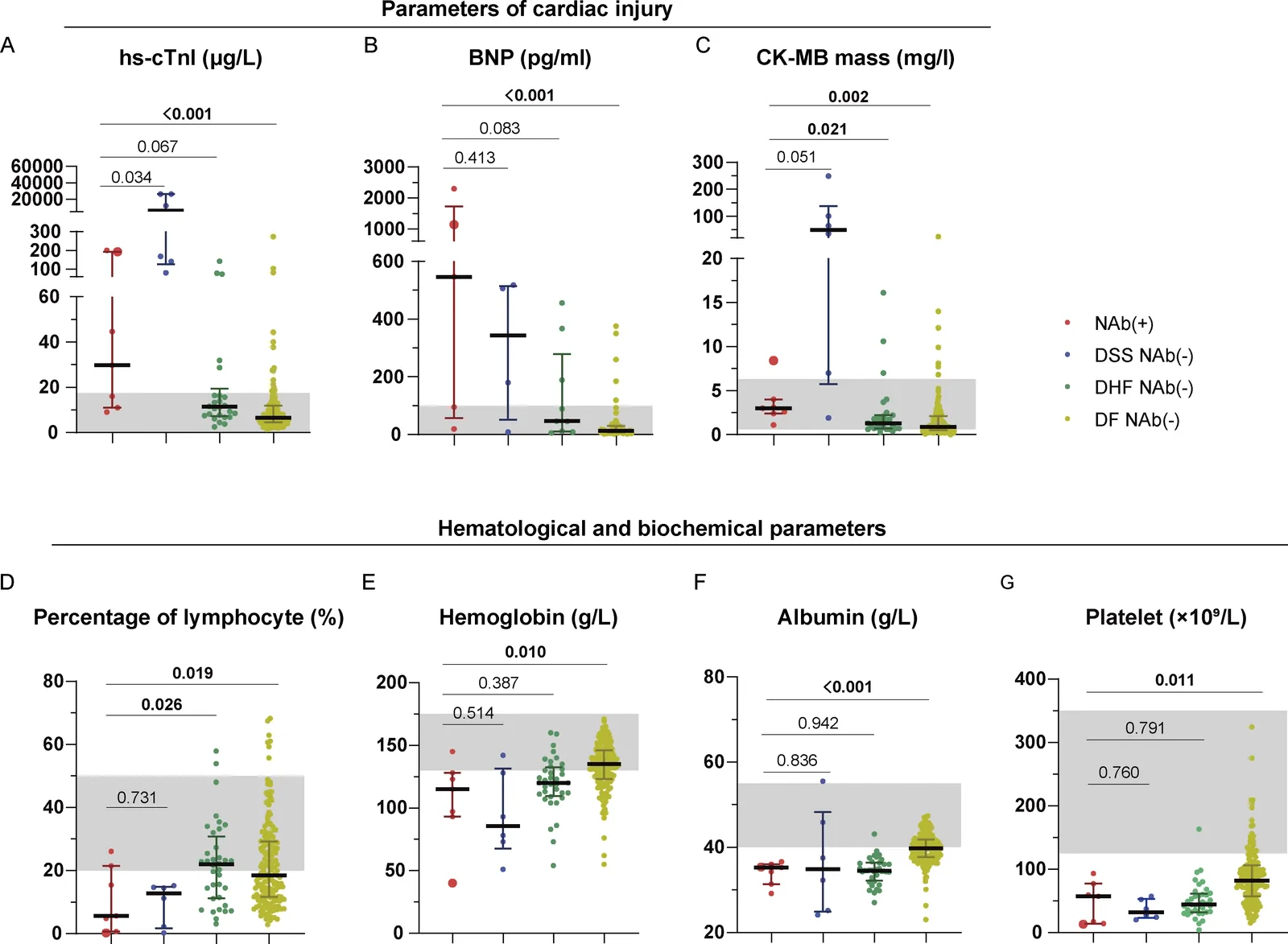
**Correlations between clinical laboratory parameters and IFN-I NAbs in dengue patients. (A–G)** Clinical laboratory parameters of NAb(+), DSS NAb(−), DHF NAb(−), and DF NAb(−) patients at the most severe stage, including parameters of cardiac injury (A, hs-cTnI; B, BNP; C, CK-MB mass). Hematological and biochemical parameters (D, percentage of lymphocytes; E, HB; F, ALB; G, platelet, PLT). The parameters of the DSS NAb(+) patient were shown as a larger red dot compared with others. Data are median with interquartile range. Statistical analysis was performed using the Mann‒Whitney U test. The gray-shaded area represents the healthy reference range for these parameters.

**Figure S2.**
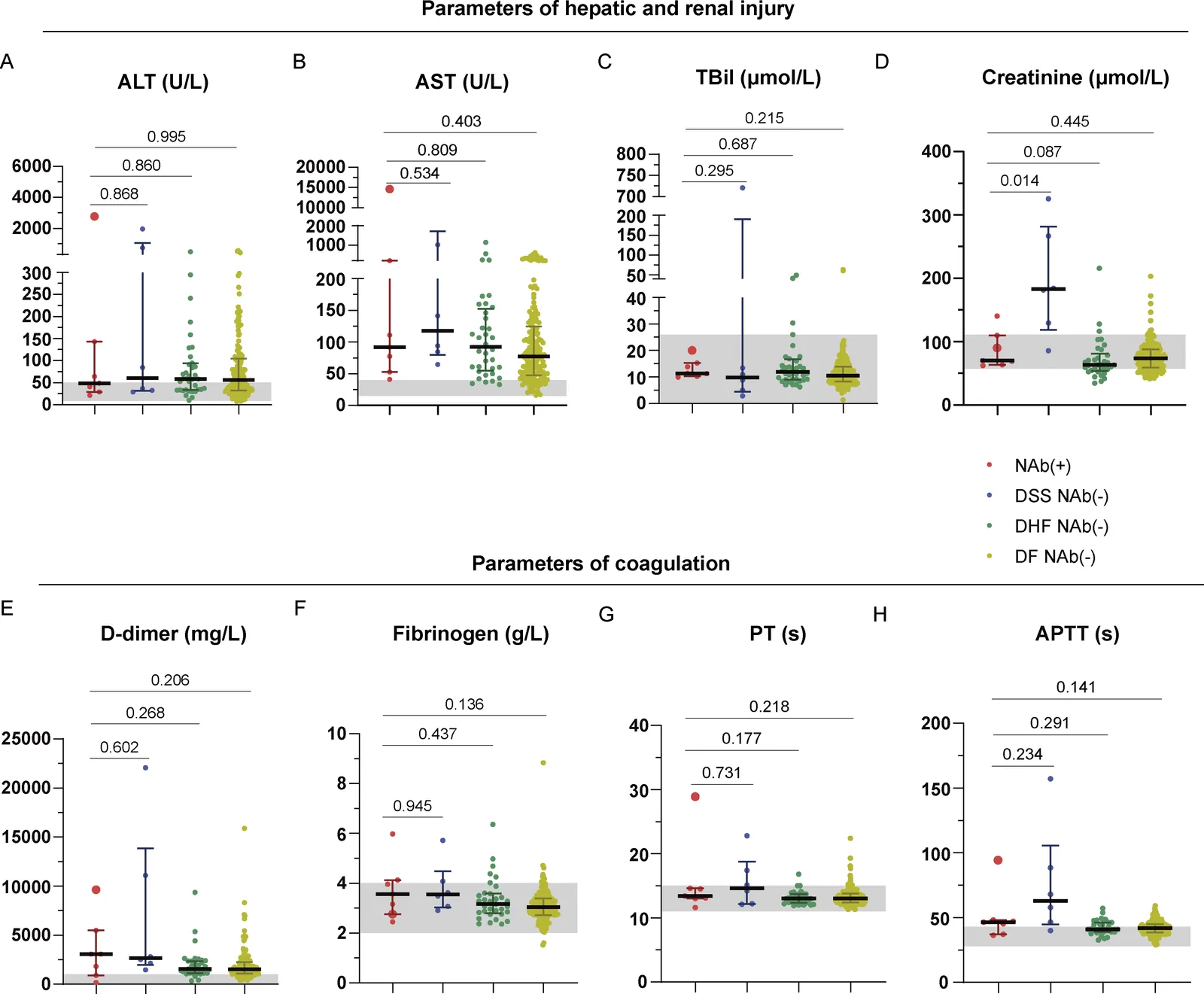
**Correlations between clinical laboratory parameters and NAbs against IFNs-I in dengue patients. (A–H)** Clinical laboratory parameters of NAb(+), DSS NAb(−), DHF NAb(−), and DF NAb(−) patients at the most severe stage, including parameters of hepatic injury (A, alanine aminotransferase, ALT; B, aspartate aminotransferase, AST), renal injury (C, total bilirubin, TBil; D, creatinine, Cr), and coagulation (E, D-dimer; F, fibrinogen, FIB; G, prothrombin time, PT; H, activated partial thromboplastin time, APTT). The parameters of the DSS NAb(+) patient were shown as a larger red dot compared with others. Data are median with interquartile range. Statistical analysis was performed using the Mann‒Whitney U test. The gray-shaded area represents the healthy reference range for these parameters.

Then we evaluated the severity of organ injury, hemorrhagic tendency, and plasma leakage by analyzing the clinical laboratory parameters of the patients during their hospitalization. For the convenience of comparison, the parameters of the only DSS NAb(+) patient were shown as a larger red dot compared with others in the NAb(+) group (Fig. 4 and Fig. S2). Laboratory parameters assessed at peak illness revealed that key biomarkers of myocardial injury, including high-sensitivity cardiac troponin I (hs-cTnI), brain natriuretic peptide (BNP), and creatine kinase-MB mass (CK-MB mass) (33), were elevated in the NAb(+) group relative to the DHF NAb(−) groups and DF NAb(−) (Fig. 4, A–C). Notably, the median BNP level—a marker reflecting heart failure—was even higher in the NAb(+) group than in the DSS NAb(−) group (Fig. 4 B). Although the medians of hematological and biochemical parameters in NAb(+) group were significantly different only from DF NAb(−) group, but the median of lymphocyte percentage, hemoglobin (HB), and ALB in the NAb(+) group were lower, approaching or falling below levels observed in the DSS NAb(−) cohort (Fig. 4, D–G), indicating associations with more severe viral infection and plasma leakage. In contrast, no significant intergroup differences were observed in the medians of parameters related to coagulation, hepatic injury, or renal injury (Fig. S2). Notably, as indicated by clinical parameters (including BNP, percentage of lymphocyte, HB, platelet, alanine aminotransferase, aspartate aminotransferase, and prothrombin time) (Fig. 4, B, D, E, and G; and Fig. S2, A, B, and G), the only DSS NAb(+) patient exhibited the most dengue severity compared with all DSS NAb(−) patients, and ultimately died according to follow-up results. Collectively, these findings suggest that the presence of NAbs is associated with increased dengue disease severity, potentially exacerbating cardiac injury and plasma leakage.

### The dynamic profiles of hematologic and myocardial enzyme parameters in dengue patients with IFN-I NAbs

**Figure 5.**
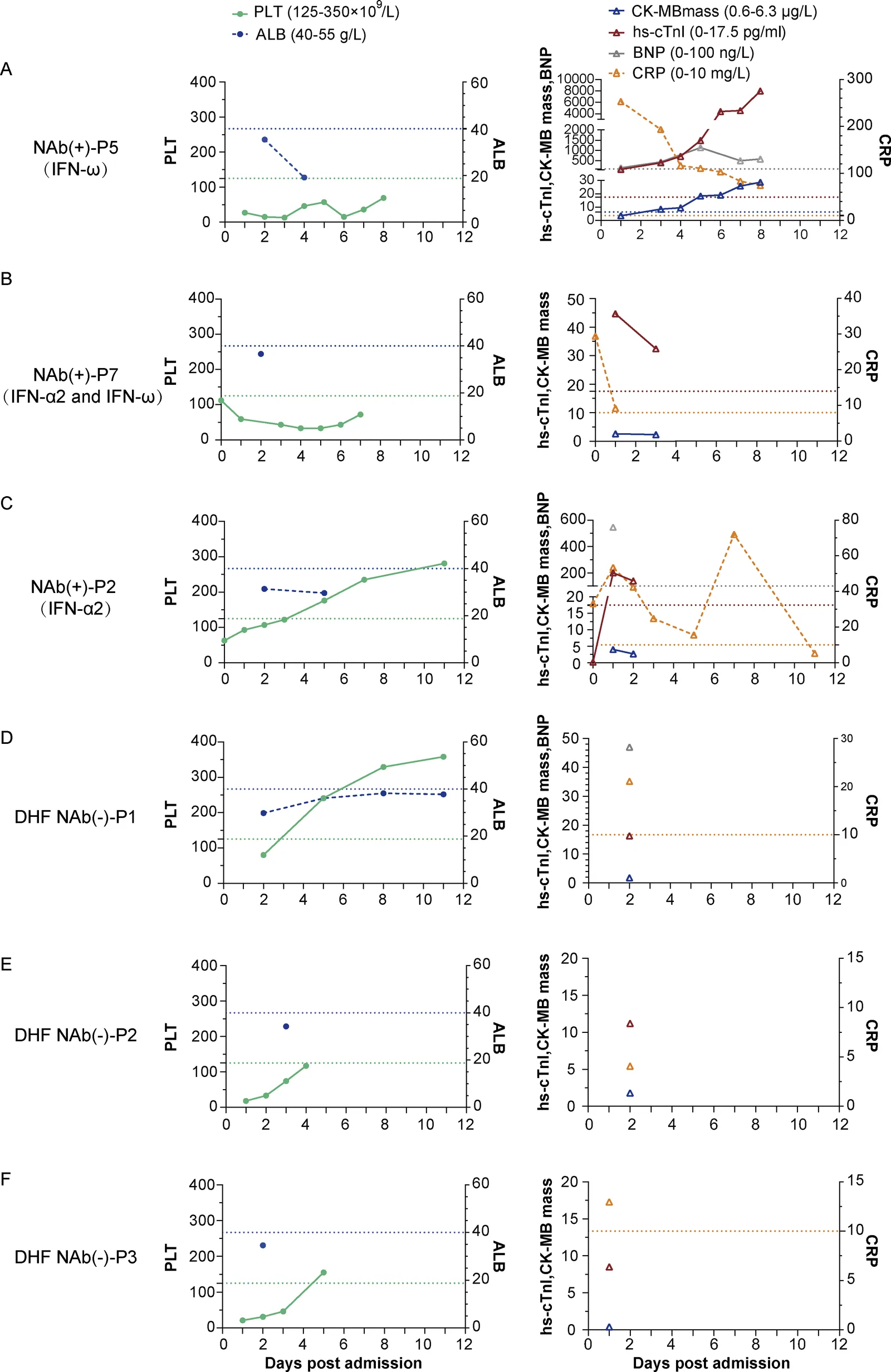
**Clinical laboratory parameters in dengue patients with IFN-I NAbs. (A–F)** The longitudinal clinical data for NAb(+) patients (A–C) and DHF NAb(−) patients (D–F). The data on day 0 represent the outpatient data or the data from other hospitals prior to hospitalization. We separately show the clinical parameters related to hemorrhage (left) and cardiac injury (right). The healthy reference ranges for all parameters are indicated in parentheses above the figure. The horizontal dashed lines in the left figure represent the lowest healthy reference values for the parameters PLT (green) and ALB (dark blue), respectively. In the right panel, the horizontal dashed lines correspond to the highest healthy reference values for CK-MB mass (blue), hs-cTnI (dark red), BNP (gray), and CRP (orange). PLT, platelet.

Finally, we investigated the dynamic profiles of platelet, ALB, myocardial enzymes, and the inflammatory marker C-reactive protein (CRP) in NAb(+) patients during hospitalization. Due to the fact that extended cardiac monitoring was not clinically indicated for most patients in the cohort, the longitudinal clinical data presented here are limited to three NAb-positive patients with relatively comprehensive documentation: one with NAbs against IFN-α2 only (Nab[+]-P2, experienced a secondary DENV infection, DHF group), one with NAbs against IFN-ω only (Nab[+]-P5, DSS group), and one with NAbs against both (Nab[+]-P7, DHF group). To be comparable, the same clinical parameters were also analyzed in a matched cohort of hospitalized DHF NAb(−) patients, including a 69-year-old patient who also experienced a secondary DENV infection (DHF Nab[−]-P3). Both P5 and P7 exhibited delayed platelet recovery and persistently low ALB levels throughout hospitalization (Fig. 5, A and B). In contrast, DHF NAb(−) patients achieved normalization of platelet counts within 5 days of admission (Fig. 5, D–F). Multiple myocardial enzyme parameters, including hs-cTnI, BNP, and CK-MB mass, were abnormal in NAb(+) patients during hospitalization. Notably, P5 demonstrated striking elevations in nearly all myocardial enzyme parameters, with peak levels of hs-cTnI (7998.8 μg/L), CK-MB mass (28.6 μg/L), and BNP (582.0 pg/ml) (Fig. 5 A). While the DHF NAb(−) patients also showed some abnormalities in CRP, this marker may reflect damage to other organs, particularly the liver. Importantly, hs-cTnI, BNP, and CK-MB mass remained within normal limits in the DHF NAb(−) group (Fig. 5, D and E). Moreover, both NAb(+)-P2 and DHF NAb(−)-P3 were elderly females who experienced secondary DENV infection, but heart failure and viral myocarditis were observed only in the NAb(+) patient. This discrepancy may be attributed to the suppression of innate immune activation by NAbs against IFNα2. Collectively, these findings indicate that patients with NAbs against IFN-I may be at an increased risk of longer hemorrhage and cardiac injury compared with those without NAbs.

## Discussion

In China, DENV transmission is largely restricted to localized outbreaks in a limited number of southern provinces, and most dengue cases in China are reported in Guangdong Province. Moreover, the DENV-infected patients in the annual endemic period are not evenly distributed. Less than 10 patients were hospitalized in 2020, 2021, and 2022 at Guangzhou Eighth People’s Hospital. Only about 20 DENV-infected patients were in 2023 and 2025. The study failed to be conducted across multiple independent cohorts. Our study presents the first systematic evaluation of NAbs against IFNs-I in a well-characterized cohort of hospitalized dengue patients and identified seven NAb-positive patients, accounting for 14.3% of the DSS and DHF groups combined. Our findings reveal that a subset of patients—primarily older individuals—harbor NAbs against IFN-α2 and/or IFN-ω, and NAbs are associated with greater dengue severity, especially increased risk of plasma leakage and cardiac injury during acute DENV infection.

Unlike the neurotropism of other flaviviruses (WNV, TBEV, POWV, and USUV), DENV infection typically induces systemic pathology through hematogenous dissemination. Hallmark clinical manifestations of severe dengue are severe hemorrhage and plasma leakage, which may directly lead to ischemic injury to the myocardium. Moreover, DENV can directly invade myocardial tissue and replicate within cardiomyocytes (34, 35). Since NAbs inhibit the anti-DENV activity of IFN-I, they may delay viral clearance in both the bloodstream and cardiac tissue. DENV infection triggers extracellular secretion of the NS1 protein, which compromises vascular endothelial barrier integrity (36, 37, 38). Collectively, these mechanisms may exacerbate hemorrhage and cardiac injury.

Notably, 57.1% (4 of 7) NAb(+) dengue patients were over 70 years old, representing 14.3% (4 of 28) of the cohort’s elderly subgroup. Advanced age is a well-established determinant of IFN-I NAb positivity in healthy individuals (15, 16, 17, 18). To test whether older dengue patients carry NAbs simply because they are older, we conducted an analysis comparing the prevalence of IFN-I NAbs across different age groups in our cohort with that reported in healthy individuals by Bastard et al. (15). Our analysis revealed that the prevalence of IFN-I NAbs was higher among elderly dengue patients than among age-matched healthy individuals, particularly in those older than 80 years, implying age is not acting as a simple determinant of more severe clinical manifestations of the disease. Our analysis suggests that screening for IFN-I NAbs in elderly dengue patients may help identify individuals at high risk for cardiac complications, thereby informing more aggressive monitoring and early intervention strategies. The potential benefit of early anti-inflammatory and cardioprotective therapy in NAb-positive patients warrants further exploration in clinical trials. Notably, severe thrombocytopenia (platelet count <20 × 10^9^/L) was observed exclusively in patients with NAbs against IFN-ω (2/2) and in one patient with NAbs against both IFN-α2 and IFN-ω (1/2). The other patients exhibited mild or moderate thrombocytopenia (Table 3), suggesting that the presence of IFN-ω NAbs may confer a higher risk of bleeding than the presence of IFN-α2. For these patients, routine monitoring of hematologic parameters is essential; platelet transfusions should be administered promptly when clinically indicated to mitigate the risk of life-threatening hemorrhagic complications.

Several limitations should be acknowledged. First, the single NAbs-positive case observed in the DSS group may be attributable to a limited sample size, thereby limiting the ability to accurately assess the effect of NAbs on severe clinical outcomes. Second, technical constraints, including the use of EDTA anticoagulant tubes and low concentrations of IFN-I in neutralization assays, may have reduced assay sensitivity and led to underestimation of NAb prevalence. Third, the cross-sectional nature of NAb detection precludes assessment of temporal dynamics and causality. Larger, multicenter studies with optimized sampling and longitudinal follow-up are needed to validate and extend our findings.

In summary, this study identifies IFN-I NAbs as a novel biomarker of increased cardiac injury in dengue patients and highlights the importance of the IFN-I pathway in modulating disease severity. Targeted screening and risk stratification based on NAb status may improve outcomes in vulnerable populations, particularly the elderly.

## Materials and methods

### Patients

We enrolled 216 consecutively admitted dengue patients (age range: 17–91 years; male: 56.9% [123/216]; female: 43.1% [93/216]) at Guangzhou Eighth People’s Hospital, Guangzhou Medical University, in 2024 in China, excluding those patients with concurrent viral co-infections. The diagnosis of DENV infection was confirmed through serological detection of DENV-specific IgM/IgG antibodies, qRT-PCR identification of viral RNA in plasma samples, and/or positive NS1 antigen testing. According to World Health Organization Guidelines for Dengue Hemorrhagic Fever Diagnosis (1997) (39), patients were stratified into three groups: DF, DHF, and DSS. The diagnosis of DF was confirmed by serological detection of DENV-specific IgM/IgG antibodies, qRT-PCR for viral RNA in plasma samples, and/or positive NS1 antigen testing. DHF was diagnosed based on the concurrent presence of four clinical criteria in patients with confirmed DF: fever, hemorrhagic tendencies, thrombocytopenia, and evidence of plasma leakage. DSS was defined as the presence of all four criteria for DHF accompanied by evidence of circulatory failure. In addition, an IgM-negative/IgG-negative or IgM-positive/IgG-negative on the blood sample drawn within 5 days of symptom onset was defined as primary infection. Secondary infection was defined as an IgM-negative/IgG-positive or an IgM-positive/IgG-positive result on the blood sample drawn within 5 days of symptom onset (40). The study was approved by Guangzhou Eighth People’s Hospital Ethics Committee (Approval number: K202502404). Written informed consent was obtained from all patients.

### Detection of NAbs using a luciferase reporter assay

To assess the neutralizing activity against IFN-α2/IFN-ω in patients’ plasma, a previously described luciferase reporter assay (19, 26) was employed. Briefly, the assay was performed in HEK-293T cells, which were co-transfected with an IFN-stimulated response element promoter-driven firefly luciferase plasmid and a constitutive *Renilla* luciferase normalization plasmid. After 24 h of incubation, cells were stimulated with high-glucose DMEM (Gibco) at 37°C for 16 h supplemented with the following components: 2% FBS (NTC), 1% plasma (for IFN-ω NAbs detection) or 2% (for IFN-α2 NAbs detection) from either patients or healthy controls (all plasma samples were heat-inactivated at 56°C for 20 min), and 100 pg/ml IFN-α2 (HY-P7022; MCE) or IFN-ω (HY-P7201; MCE). Luciferase activity was quantified after lysis using a dual-reporter system (11402ES80; Yeasen), where firefly luciferase signals were normalized to *Renilla* luciferase signals. The neutralization capacity of each sample was calculated as the percentage of luciferase activity relative to non-neutralized controls. Samples were defined as neutralizing activity if its IFN-induced luminescence was <15% of the median IFN-induced luminescence in control wells.

### DENV-1 isolated from patients

Following DENV serotype screening and quantification via qRT-PCR, plasma samples from patients with higher DENV-1 viral loads (cycle threshold [CT] <20) were selected for viral isolation. Add 10 μl of selected plasma to C6/36 cell monolayers and maintain in RPMI-1640 supplemented with 2% FBS. After three serial passages, viral titers were quantified by focus-forming assay.

### Assessment of antiviral activity of IFN against DENV-1 in the presence of dengue patient plasma

To evaluate the impact of NAbs on IFN antiviral activity, Huh7 cells were pretreated with medium containing 1 ng/ml IFN-α2 or IFN-ω and 2% patient plasma, then infected with DENV-1 at a multiplicity of infection of 0.5. After 2 h of viral adsorption, the culture medium was removed and replaced with complete medium. At 24 h after infection, both the Huh-7 cells and culture supernatants were harvested for DENV RNA quantification via qRT-PCR.

### Statistical analysis

All statistical analyses were performed using R software (version 4.3.3) and GraphPad Prism (version 9.5). Continuous variables were analyzed using Student’s *t* test or Mann–Whitney U test as appropriate; categorical variables were evaluated using chi-square (when all expected frequencies ≥5) or Fisher’s exact test (for sparse data with expected frequencies <5). Firth’s bias-corrected logistic regression was used to assess the association between clinical characteristics and IFN-I NAbs with adjusted ORs and 95% CIs. A P value <0.05 was considered statistically significant.

### Online supplemental material

Fig. S1 shows effects of different proportions of plasma (collected by EDTA-anticoagulated tubes) on IFN-I activation. Fig. S2 shows correlations between clinical laboratory parameters and NAbs against IFNs-I in dengue patients.

## Data Availability

All data supporting the findings of this study are available within the main text and supplemental material.

## References

[1] Bhatt, S., P.W. Gething, O.J. Brady, J.P. Messina, A.W. Farlow, C.L. Moyes, J.M. Drake, J.S. Brownstein, A.G. Hoen, O. Sankoh, . 2013. The global distribution and burden of dengue. Nature. 496:504–507. 10.1038/nature12060 23563266 PMC3651993

[2] Shepard, D.S., E.A. Undurraga, Y.A. Halasa, and J.D. Stanaway. 2016. The global economic burden of dengue: A systematic analysis. Lancet Infect. Dis. 16:935–941. 10.1016/S1473-3099(16)00146-8 27091092

[3] World Health Organization . 2009. Dengue: Guidelines for Diagnosis, Treatment, Prevention and Control. New edition. World Health Organization, Geneva, Switzerland. Available from: https://www.who.int/publications/i/item/9789241547871 23762963

[4] World Health Organization . 2022. National guideline for clinical management of dengue. Available from: https://www.who.int/timorleste/publications/national-guideline-for-clinical-management-of-dengue-2022

[5] Hernandez, N., G. Bucciol, L. Moens, J. Le Pen, M. Shahrooei, E. Goudouris, A. Shirkani, M. Changi-Ashtiani, H. Rokni-Zadeh, E.H. Sayar, . 2019. Inherited IFNAR1 deficiency in otherwise healthy patients with adverse reaction to measles and yellow fever live vaccines. J. Exp. Med. 216:2057–2070. 10.1084/jem.20182295 31270247 PMC6719432

[6] Duncan, C.J., S.M. Mohamad, D.F. Young, A.J. Skelton, T.R. Leahy, D.C. Munday, K.M. Butler, S. Morfopoulou, J.R. Brown, M. Hubank, . 2015. Human IFNAR2 deficiency: Lessons for antiviral immunity. Sci. Transl. Med. 7:307ra154. 10.1126/scitranslmed.aac4227 PMC4926955 26424569

[7] Dupuis, S., E. Jouanguy, S. Al-Hajjar, C. Fieschi, I.Z. Al-Mohsen, S. Al-Jumaah, K. Yang, A. Chapgier, C. Eidenschenk, P. Eid, . 2003. Impaired response to interferon-α/β and lethal viral disease in human STAT1 deficiency. Nat. Genet. 33:388–391. 10.1038/ng1097 12590259

[8] Vairo, D., L. Tassone, G. Tabellini, N. Tamassia, S. Gasperini, F. Bazzoni, A. Plebani, F. Porta, L.D. Notarangelo, S. Parolini, . 2011. Severe impairment of IFN-γ and IFN-α responses in cells of a patient with a novel STAT1 splicing mutation. Blood. 118:1806–1817. 10.1182/blood-2011-01-330571 21772053

[9] Chapgier, A., X.F. Kong, S. Boisson-Dupuis, E. Jouanguy, D. Averbuch, J. Feinberg, S.-Y. Zhang, J. Bustamante, G. Vogt, J. Lejeune, . 2009. A partial form of recessive STAT1 deficiency in humans. J. Clin. Invest. 119:1502–1514. 10.1172/JCI37083 19436109 PMC2689115

[10] Kong, X.F., M. Ciancanelli, S. Al-Hajjar, L. Alsina, T. Zumwalt, J. Bustamante, J. Feinberg, M. Audry, C. Prando, V. Bryant, . 2010. A novel form of human STAT1 deficiency impairing early but not late responses to interferons. Blood. 116:5895–5906. 10.1182/blood-2010-04-280586 20841510 PMC3031383

[11] Moens, L., L. Van Eyck, D. Jochmans, T. Mitera, G. Frans, X. Bossuyt, P. Matthys, J. Neyts, M. Ciancanelli, S.-Y. Zhang, . 2017. A novel kindred with inherited STAT2 deficiency and severe viral illness. J. Allergy Clin. Immunol. 139:1995–1997.e9. 10.1016/j.jaci.2016.10.033 28087227

[12] Fuchs, S., P. Kaiser-Labusch, J. Bank, S. Ammann, A. Kolb-Kokocinski, C. Edelbusch, H. Omran, and S. Ehl. 2016. Tyrosine kinase 2 is not limiting human antiviral type III interferon responses. Eur. J. Immunol. 46:2639–2649. 10.1002/eji.201646519 27615517

[13] Sarrafzadeh, S.A., M. Mahloojirad, J.L. Casanova, M. Badalzadeh, J. Bustamante, S. Boisson-Dupuis, Z. Pourpak, M. Nourizadeh, and M. Moin. 2020. A new patient with inherited TYK2 deficiency. J. Clin. Immunol. 40:232–235. 10.1007/s10875-019-00713-5 31713088 PMC7218688

[14] Kreins, A.Y., M.J. Ciancanelli, S. Okada, X.F. Kong, N. Ramírez-Alejo, S.S. Kilic, J. El Baghdadi, S. Nonoyama, S.A. Mahdaviani, F. Ailal, . 2015. Human TYK2 deficiency: Mycobacterial and viral infections without hyper-IgE syndrome. J. Exp. Med. 212:1641–1662. 10.1084/jem.20140280 26304966 PMC4577846

[15] Bastard, P., A. Gervais, T. Le Voyer, J. Rosain, Q. Philippot, J. Manry, E. Michailidis, H.-H. Hoffmann, S. Eto, M. Garcia-Prat, . 2021. Autoantibodies neutralizing type I IFNs are present in ∼4% of uninfected individuals over 70 years old and account for ∼20% of COVID-19 deaths. Sci. Immunol. 6:eabl4340. 10.1126/sciimmunol.abl4340 34413139 PMC8521484

[16] Bastard, P., S.E. Vazquez, J. Liu, M.T. Laurie, C.Y. Wang, A. Gervais, T. Le Voyer, L. Bizien, C. Zamecnik, Q. Philippot, . 2023. Vaccine breakthrough hypoxemic COVID-19 pneumonia in patients with auto-Abs neutralizing type I IFNs. Sci. Immunol. 8:eabp8966. 10.1126/sciimmunol.abp8966 35857576 PMC9210448

[17] Puel, A., P. Bastard, J. Bustamante, and J.L. Casanova. 2022. Human autoantibodies underlying infectious diseases. J. Exp. Med. 219:e20211387. 10.1084/jem.20211387 35319722 PMC8952682

[18] Zhang, Q., P. Bastard, C.H.G. Effort, A. Cobat, and J.L. Casanova. 2022. Human genetic and immunological determinants of critical COVID-19 pneumonia. Nature. 603:587–598. 10.1038/s41586-022-04447-0 35090163 PMC8957595

[19] Gervais, A., F. Rovida, M.A. Avanzini, S. Croce, A. Marchal, S.C. Lin, A. Ferrari, C.W. Thorball, O. Constant, T. Le Voyer, . 2023. Autoantibodies neutralizing type I IFNs underlie West Nile virus encephalitis in approximately 40% of patients. J. Exp. Med. 220:e20230661. 10.1084/jem.20230661 37347462 PMC10287549

[20] Barzaghi, F., C. Visconti, G.B. Pipitone, S. Bondesan, G. Molli, S. Giannelli, C. Sartirana, V. Lampasona, E. Bazzigaluppi, C. Brigatti, . 2025. Severe West Nile virus and severe acute respiratory syndrome coronavirus 2 infections in a patient with thymoma and anti-type I interferon antibodies. J. Infect. Dis. 231:e206–e212. 10.1093/infdis/jiae321 38976510 PMC11793036

[21] Gervais, A., A. Marchal, S. Boucherit, A. Abi Haidar, L. Bizien, A. Yalcinkaya, E. Sandström, X.-F. Kong, E. Jacquemin, O. Bernard, . 2026. Autoantibodies neutralizing type I IFNs in patients with fulminant herpes simplex virus hepatitis. J. Exp. Med. 223:e20251760. 10.1084/jem.20251760 41348319 PMC12679989

[22] Bastard, P., L.B. Rosen, Q. Zhang, E. Michailidis, H.H. Hoffmann, Y. Zhang, K. Dorgham, Q. Philippot, J. Rosain, V. Béziat, . 2020. Autoantibodies against type I IFNs in patients with life-threatening COVID-19. Science. 370:eabd4585. 10.1126/science.abd4585 32972996 PMC7857397

[23] Zhang, Q., P. Bastard, A. Bolze, E. Jouanguy, S.Y. Zhang, C.H.G. Effort, A. Cobat, L.D. Notarangelo, H.C. Su, L. Abel, and J.-L. Casanova. 2020. Life-threatening COVID-19: Defective interferons unleash excessive inflammation. Med. 1:14–20. 10.1016/j.medj.2020.12.001 33363283 PMC7748410

[24] van der Wijst, M.G.P., S.E. Vazquez, G.C. Hartoularos, P. Bastard, T. Grant, R. Bueno, D.S. Lee, J.R. Greenland, Y. Sun, R. Perez, . 2021. Type I interferon autoantibodies are associated with systemic immune alterations in patients with COVID-19. Sci. Transl. Med. 13:eabh2624. 10.1126/scitranslmed.abh2624 34429372 PMC8601717

[25] Koning, R., P. Bastard, J.L. Casanova, M.C. Brouwer, D. van de Beek, and with the Amsterdam U.M.C. COVID-19 Biobank Investigators. 2021. Autoantibodies against type I interferons are associated with multi-organ failure in COVID-19 patients. Intensive Care Med. 47:704–706. 10.1007/s00134-021-06392-4 33835207 PMC8034036

[26] Shi, D., J. Chen, M. Zhao, Y. Tang, C. Zhao, Y. Jin, D. Tian, Y. Liao, X. Wang, W. Wang, . 2024. Prevalence of neutralizing autoantibodies against type I interferon in a multicenter cohort of severe or critical COVID-19 cases in Shanghai. J. Clin. Immunol. 44:80. 10.1007/s10875-024-01683-z 38462559 PMC10925575

[27] Alotaibi, F., N.K. Alharbi, L.B. Rosen, A.Y. Asiri, A.M. Assiri, H.H. Balkhy, M. Al Jeraisy, Y. Mandourah, S. AlJohani, S. Al Harbi, . 2023. Type I interferon autoantibodies in hospitalized patients with Middle East respiratory syndrome and association with outcomes and treatment effect of interferon beta-1b in MIRACLE clinical trial. Influenza Other Respir. Viruses. 17:e13116. 10.1111/irv.13116 36960162 PMC10028524

[28] Gervais, A., A. Marchal, A. Fortova, M. Berankova, L. Krbkova, M. Pychova, J. Salat, S. Zhao, N. Kerrouche, T. Le Voyer, . 2024. Autoantibodies neutralizing type I IFNs underlie severe tick-borne encephalitis in ∼10% of patients. J. Exp. Med. 221:e20240637. 10.1084/jem.20240637 39316018 PMC11448868

[29] Zhang, Q., A. Pizzorno, L. Miorin, P. Bastard, A. Gervais, T. Le Voyer, L. Bizien, J. Manry, J. Rosain, Q. Philippot, . 2022. Autoantibodies against type I IFNs in patients with critical influenza pneumonia. J. Exp. Med. 219:e20220514. 10.1084/jem.20220514 36112363 PMC9485705

[30] Zhang, Q., T.S. Conrad, M. Moncada-Velez, K. Jiang, A. Cupic, J. Eaton, K. Hutchinson, A. Gervais, R. Chen, A. Puel, . 2026. Autoantibodies neutralizing type I IFNs in a fatal case of H5N1 avian influenza. J. Exp. Med. 223:e20251962. 10.1084/jem.20251962 41348320 PMC12679982

[31] Gervais, A., P. Bastard, L. Bizien, C. Delifer, P. Tiberghien, C. Rodrigo, F. Trespidi, M. Angelini, G. Rossini, T. Lazzarotto, . 2024. Auto-Abs neutralizing type I IFNs in patients with severe Powassan, Usutu, or Ross River virus disease. J. Exp. Med. 221:e20240942. 10.1084/jem.20240942 39485284 PMC11533500

[32] Bastard, P., E. Michailidis, H.H. Hoffmann, M. Chbihi, T. Le Voyer, J. Rosain, Q. Philippot, Y. Seeleuthner, A. Gervais, M. Materna, . 2021. Auto-antibodies to type I IFNs can underlie adverse reactions to yellow fever live attenuated vaccine. J. Exp. Med. 218:e20202486. 10.1084/jem.20202486 33544838 PMC7871457

[33] Elamm, C., D. Fairweather, and L.T. Cooper. 2012. Pathogenesis and diagnosis of myocarditis. Heart. 98:835–840. 10.1136/heartjnl-2012-301686 22442199 PMC12767484

[34] Kularatne, S.A., I.V. Imbulpitiya, R.A. Abeysekera, R.N. Waduge, R.P. Rajapakse, and K.G. Weerakoon. 2014. Extensive haemorrhagic necrosis of liver is an unpredictable fatal complication in dengue infection: A postmortem study. BMC Infect. Dis. 14:141. 10.1186/1471-2334-14-141 24628767 PMC4008311

[35] Kangussu, L.M., V.V. Costa, V.C. Olivon, C.M. Queiroz-Junior, A.N.S. Gondim, M.B. Melo, D. Reis, N. Nóbrega, N. Araújo, M.A. Rachid, . 2022. Dengue virus infection induces inflammation and oxidative stress on the heart. Heart. 108:388–396. 10.1136/heartjnl-2020-318912 34049953

[36] Pan, P., G. Li, M. Shen, Z. Yu, W. Ge, Z. Lao, Y. Fan, K. Chen, Z. Ding, W. Wang, . 2021. DENV NS1 and MMP-9 cooperate to induce vascular leakage by altering endothelial cell adhesion and tight junction. PLoS Pathog. 17:e1008603. 10.1371/journal.ppat.1008603 34310658 PMC8341711

[37] Beatty, P.R., H. Puerta-Guardo, S.S. Killingbeck, D.R. Glasner, K. Hopkins, and E. Harris. 2015. Dengue virus NS1 triggers endothelial permeability and vascular leak that is prevented by NS1 vaccination. Sci. Transl. Med. 7:304ra141. 10.1126/scitranslmed.aaa3787 26355030

[38] Glasner, D.R., K. Ratnasiri, H. Puerta-Guardo, D.A. Espinosa, P.R. Beatty, and E. Harris. 2017. Dengue virus NS1 cytokine-independent vascular leak is dependent on endothelial glycocalyx components. PLoS Pathog. 13:e1006673. 10.1371/journal.ppat.1006673 29121099 PMC5679539

[39] World Health Organization . 1997. Dengue Guidelines, for Diagnosis, Treatment, Prevention and Control. World Health Organization, Geneva, Switzerland.

[40] Changal, K.H., A.H. Raina, A. Raina, M. Raina, R. Bashir, M. Latief, T. Mir, and Q.H. Changal. 2016. Differentiating secondary from primary dengue using IgG to IgM ratio in early dengue: An observational hospital based clinico-serological study from north India. BMC Infect. Dis. 16:715. 10.1186/s12879-016-2053-6 27894268 PMC5127094

